# An explainable deep learning approach for stock market trend prediction

**DOI:** 10.1016/j.heliyon.2024.e40095

**Published:** 2024-11-05

**Authors:** Dost Muhammad, Iftikhar Ahmed, Khwaja Naveed, Malika Bendechache

**Affiliations:** a CRT-AI and ADAPT Research Centres, School of Computer Science, University of Galway, Ireland; b Department of Software Engineering, University of Europe for Applied Sciences, Potsdam, Germany; c Swedish Center for Digital Innovation, University of Gothenburg, Sweden; d ADAPT Research Centre, School of Computer Science, University of Galway, Ireland

**Keywords:** Explainable AI, Deep neural network, Stock market trend prediction, Time series prediction, Machine learning

## Abstract

Given the intricate nature of stock forecasting as well as the inherent risks and uncertainties, analysis of market trends is necessary to capitalize on optimal investment opportunities for profit maximization and timely disinvestment for loss minimization. In this work, we propose a deep learning model for predicting five distinct stock market trends: upward, downward, double top, rounded bottom, and rounded top. The proposed model surpasses common benchmarks, including support vector machine, random forest, and logistic regression, achieving an average accuracy of 94.9%, compared to 85.7% for random forest, 60.07% for support vector machine, and 52.45% for logistic regression. Furthermore the proposed model excels in F1-score, with a 94.85% performance, compared to 77.95% for random forest, 21.02% for support vector machine and 46.23% for logistic regression, across four real world diverse datasets. Additionally, we employ explainable AI (XAI) techniques, SHAP and LIME, to enhance interpretability, enabling stakeholders to understand the key factors driving predictions. The SHAP analysis reveals the top 10 most important/influential features, enabling feature reduction while maintaining performance. Interestingly, while accuracy slightly decreases with top 10 features, precision, recall, and F1-score improve, suggesting a trade-off between comprehensiveness and performance. These results demonstrate the potential for practical application in financial decision-making, providing a balance between interpretability and predictive power that can support investors in risk management and strategic planning.

## 1 Introduction

Stock market is an important indicator of a country's economic health, being regarded as the lynchpin of the global financial system. For this reason, the governments, investors and industry stakeholders follow and monitor the fluctuations in stock markets [1]. These markets have a potential of high payback as compared to the bank investments and bonds, if managed prudently. In 2022, the market capitalization of stock markets across the globe risen to 70.75 Trillion USD, a resounding testament of the profound significance of stock markets in global financial system [2].

In finance, researchers have extensively explored stock trend prediction, acknowledging its importance in shaping stock trading strategies, mitigating investment risks, and achieving favourable returns [1]. Notably, investigations in this area have revealed correlations between various factors, including firm characteristics such as age, size, and financial strengths, as well as broader economic conditions, public sentiments, and political landscapes, elucidating their associations with stock behaviour. The complex interaction and association among these various factors causes stock prices to fluctuate, ultimately shaping the overall trends seen in the stock markets. Navigating this dynamic often resembles a zero-sum game, as unexpected trends can jeopardize entire investments. Consequently, strategic planning grounded in trend identification becomes imperative, necessitating meticulous consideration of these dynamic factors to effectively mitigate risks and devise enhanced investment plans. Stock market investing has experienced a significant transformation with the emergence of disruptive technologies and the internet revolution. The exchange of securities has been transformed by the use of virtual assistants and online trading platforms, which uses a variety of algorithms that outperform conventional techniques and give more profitability.

In recent years, advancements in artificial intelligence (AI), machine learning (ML) and deep learning (DL) have increasingly been applied to financial markets, driven by their ability to handle large volumes of data and detect complex patterns [3], [4], [5]. ML and DL, have demonstrated valuable performance in financial stock market due to their capacity to capture non-linear relationships and process unstructured data [6]. These advancements provide a more nuanced understanding of market dynamics and enhance prediction accuracy, placing AI at the forefront of financial decision-making. Researchers have mostly focused on predicting the precise value of stock prices (stock price prediction), overlooking the crucial aspect of classifying and predicting the broader trends in stock. While several approaches, such as technical analysis, time series analysis, and ML techniques [2], [7], [8] have been explored, emphasis on multi-class trends in stock direction has been limited [9], [10]. A scant number of studies that have made efforts to address this gap have encountered difficulties such as dataset constraints, binary categorization of upward and downward trends, as well as problems with efficiency, feature detection, and structural optimisation [11], [12], [13], [14], [15]. Moreover, ML for stock market trends prediction presents several unique challenges, which must be considered to build robust models. One of the most prominent issues is overfitting [16], where models may perform well on training data but fail to generalize to unseen data due to the complex and often non-stationary nature of financial markets. Market volatility is another critical challenge, as sudden, unpredictable market swings can reduce the effectiveness of models trained on historical data [17]. Additionally, the quality and availability of datasets play a pivotal role; stock data often contains noise, missing values, or biased information, which can negatively impact model performance. In the same vein, due to the interpretability issue of common ML techniques, the aspects, variables, and patterns driving the model's predictions in a dynamic and complex stock market environment is near to impossible to be properly comprehended.

**Figure 1:**
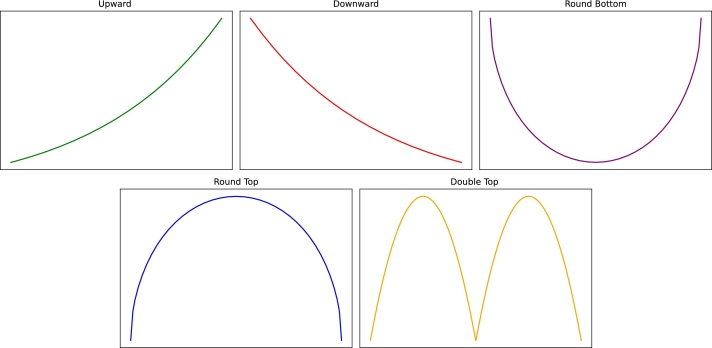
The five trends considered in this paper.

In response to the gap, this research proposes a DL technique for stock market's trends prediction. In contrast to the existing works which mainly focus on limited trends, we consider 5 trends namely upward, downward, double top, rounded bottom, and rounded top [18]. Fig. 1 is a graphical representation of the aforementioned five trends. Note that this is an ideal representation and the actual trends representation may vary. These specific trends were selected due to their significance in financial technical analysis. Upward and downward trends represent basic market movements crucial for identifying bullish or bearish momentum, while double top, rounded bottom, and rounded top are well-known reversal patterns often used to predict shifts in market direction, making them valuable for practical trading decisions.

The formal research question for this study is: “Can a DL model accurately predict multiple distinct stock market trends (upward, downward, double top, rounded bottom, and rounded top), and how can Explainable Artificial Intelligence (XAI) enhance the interpretability of these predictions for financial stakeholders?” This research question frames the central investigation and aims to fill a significant gap in the existing literature. We compare our propose approach with support vector machine (SVM), random forest (RF) classifier, and Logistic Regression (LR) on diverse datasets including S&P500, DAX30, Nikie225 and FTSE considering various performance metrics. Further, to improve the understandability of complicated stock market prediction models, this study uses XAI methodologies, concentrating on SHapley Additive exPlanations (SHAP) [19], [20], [21], [22] and Local Interpretable Model-agnostic Explanations (LIME) [23], [24], [25], [26]. We quantify the contribution of each feature to the SHAP model's predictions to highlight the relative influence of various variables on the final outcome. To make well-informed decisions, this helps investors and analysts identify the key variables that influence the model's outcomes. The LIME system also produced locally understandable explanations for individual forecasts, enabling stakeholders to understand how particular examples are influenced by input features. The main contributions of this study are threefold:

• The development of a deep learning model that accurately predicts five distinct stock market trends, addressing a gap in the literature on multi-class stock trend classification.
• A thorough comparison with benchmark ML models (RF, SVM, and LR) across multiple real-world datasets, demonstrating superior predictive performance.
• The integration of XAI techniques (SHAP and LIME), providing stakeholders with an interpretable framework for understanding model predictions, thereby improving trust and aiding strategic financial decision-making.

This contribution improves dependability of AI-driven predictions and enables stakeholders to make more strategic decisions in a tumultuous stock market environment.

The rest of the paper is organized as follows. Section 2 provides a succinct overview of the existing literature. Section 3 describes the proposed methodology for the study, Section 4 reports the results, findings and discussion and finally Section 5 sum up the conclusion.

## 2 Literature review

The existing body of research on predicting stock prices highlights the classification of multiple label trends as an unresolved challenge [7], [8], [9], [10], [11], [12], [13], [14], [15]. The authors of [36] implemented logistic regression model for forecasting of stock prices. The authors used LASSO to predict price of the stock based on daily data of Gold Sachs Group Inc., for 1999-2014. The contributions are restricted to price prediction and trends are not considered. The researchers in [8], used support vector machine to predict stock prices in different financial-markets. Although the authors applied the proposed model on Chinese, Brazilian and American stock markets, they did not predict the stock prices efficiently. Gong et al. [11] utilized logistic regression to forecast the trends of stock prices in the following month using data from the current month. However, their evaluation of the model was not exhaustive, and some of the feature variables did not yield significant results.

Khan et al. [7] assessed the performance of a regression-based model across major stock exchanges and top companies. They used extensive historical data from Yahoo! Finance to address the complexity of the stock market and demonstrate strong forecasting accuracy. The results, presented through Mean Absolute Error (MAE) and Root Mean Square Error (RMSE), indicate favourable outcomes. Zhong et al. [27] addressed the challenge of predicting daily stock market returns by proposing a comprehensive data mining process utilizing three dimensionality reduction techniques. By applying artificial neural networks to the transformed datasets, they achieve improved classification accuracy, particularly when combined with principal component analysis, leading to higher risk-adjusted profits in trading strategies compared to other models. Lee [12] developed a prediction model using support vector machine and a hybrid feature selection method, F-score and Supported Sequential Forward Search, to forecast stock market trends. The author compared the performance of the proposed model with back-propagation neural network and three other feature selection methods, demonstrating support vector machine superiority in accuracy. The findings suggest that support vector machine combined with F-score and Supported Sequential Forward Search shows better performance in stock trend prediction methods

Lin et al. [14] introduced a support vector machine-based approach for stock market trend prediction, comprising feature selection and a prediction model. By employing a correlation-based support vector machine (SVM) filter for feature selection and a quasi-linear SVM for prediction, the method demonstrated improved generalization performance and identifies valuable stock indicators, as evidenced by experiments on Taiwan stock market datasets. Ratto et al. [15] introduced a new approach that utilized technical analysis to forecast the directional movements of NASDAQ's top stocks, addressing the challenge of skewed classes with data balancing techniques. However, the model achieved an accuracy of 62% on binary classification problem. Trends prediction is also studied by [28] whereby they discussed different machine learning algorithms namely multi layer perceptrons, naive bayes, support vector machines, recurrent neural network, long short term memory and decision trees for trends prediction. The authors focused on the optimum combination of different algorithms for a problem where they used trend prediction as a subject rather than focusing on multi-label classification and accuracy. The authors of [29] applied Bayesian-regularized artificial neural network to predict the financial market behaviour. Technical indicators and stock market trends were used as inputs to predict the cost of individual stock. However, the number of indicators were limited and the classification is also binary. The study [30] presented various machine learning techniques such as CHAID, CART, SVM and ANN on BSE SENSEX data-set for prediction and analyzing the stock trends. However, they used a single data-set and the classification is also binary. The researchers of [31] designed a recurrent-CNN for short term stock market trends prediction using financial news. The proposed approach failed using financial knowledge for model optimization to predict the stock trends. The authors of [32] developed a method to predict the movement of daily price using the combination of various techniques such as adaptive neuro-fuzzy-inference systems, SVM and ANN using data analytics. The authors tested the proposed model on 8 years data of only Istanbul stock index.

Due to the complexity of machine learning models and their applications in domains such as finance, where they are used to forecast stock prices, market directions, and returns, explainable artificial intelligence (XAI) has attracted interest. Ohan et al. [33] used gradient boosting decision trees to predict stock prices. To make the model predictions easier to understand, the authors incorporated Shapley values, a popular XAI method. In other works, [34] focused on the Local Interpretable Model-agnostic Explanations (LIME) method to predict stock market direction using machine learning techniques.

LIME is used to produce locally accurate justifications for certain forecasts, assisting in comprehending the model's choices. To clarify how to interpret the decision-making process of the model, the authors in [35] expanded the application of machine learning to forecast stock market returns. Their work demonstrated the application of different ML techniques, helping improve predictions of stock market returns.

The existing literature presented in Table 1 on stock market prediction has primarily focused on binary classification of trends, which is insufficient for capturing the complexity of financial markets. Approaches namely SVM and RF often fail to generalize well to multi-class trend prediction due to their limitations in handling non-linear relationships and sequential dependencies inherent in stock market data. While SVM-based models have shown some promise in binary classification, they struggle to adequately capture the subtle variations and intricate patterns required for accurate multi-class classification, as evidenced by limited accuracy and generalization across diverse datasets [12], [14]. Additionally, regression-based models often focus on price prediction rather than trend forecasting, overlooking the need for multi-class trend classification in financial contexts [11], [7]. Recent advancements in DL, such as RNNs and Long Short-Term Memory (LSTM) models, have shown potential in time-series forecasting by capturing temporal dependencies in data [37]. However, these models often struggle with long-term dependencies and require complex architectures to achieve competitive accuracy [38], [39]. In contrast, our proposed Deep Neural Network (DNN) addresses these limitations by leveraging its ability to model complex, non-linear patterns and handle multi-class classification problems effectively. Furthermore, we integrate XAI techniques namely SHAP and LIME, which provide interpretability, allowing stakeholders to understand the key factors influencing predictions. This combination not only improves prediction accuracy but also enhances model transparency and trustworthiness, differentiating our approach from previous works that lack sufficient explainability.

**Table 1:** Comparison of our proposed framework with extant literature.

Authors	Dataset	Data time frame	Approaches	Evaluation metrics	Trends/Price prediction	No of trends	Integration with XAI
[8]	Group Inc.	1999-2014	LR, LASSO	RMSE	Price	0	No

[11]	Brazilian, American and Chinese stocks	2002-2017	SVM	RMSE	Price	0	No
[7]	Shenzhen Development stock	2007	LR	Accuracy	Trends	2	No
[27]	New York, London, NASDAQ and Karachi	1998-2018	Regression-based	MAE, RMSE	Price	0	No
[12]	S&P 500	2003-2013	PCA, ANN	Accuracy	Price	0	No
[14]	NASDAQ	Not-mentioned	SVM	Accuracy	Trends	2	
[15]	Taiwan	2008-2012	SVM	Accuracy	Trends	2	No
[28]	NASDAQ	Not-mentioned	SVM	Accuracy	Trends	2	No
[29]	China	2008-2015	MLP, RNN, LSTM, NB, and DT	Accuracy	Trends	2	No
[30]	Microsoft Corp. and Goldman Sachs Group Inc. stock	2010-2012	Bayesian-ANN	MAPE	Price	0	No
[31]	BSE SENSEX	2012-2018	ANN, SVM	MAE, MAPE	Trends	2	No
[32]	Apple, Google, and Microsoft	2015-2016	Recurrent-CNN	Accuracy	Trends	2	No
[33]	Istanbul, NASDAQ	2007-2014	SVM, ANN	Accuracy	Trends	2	No
[34]	S&P 500	2020	Gradient boosting	Avg-precision	Price	0	Yes (LIME)
[35]	SP500, NI225, XU100, KOSPI	10 years	ANN	Accuracy	Price	0	Yes (LIME)
**Our proposed**	S&P 500, DAX30, FTSE100, Nikkie225	1990-2022	DNN	Accuracy, Precision, Recall, and F1 score	Trends	5	Yes (LIME, SHAP)

## 3 Proposed methodology

**Figure 2:**
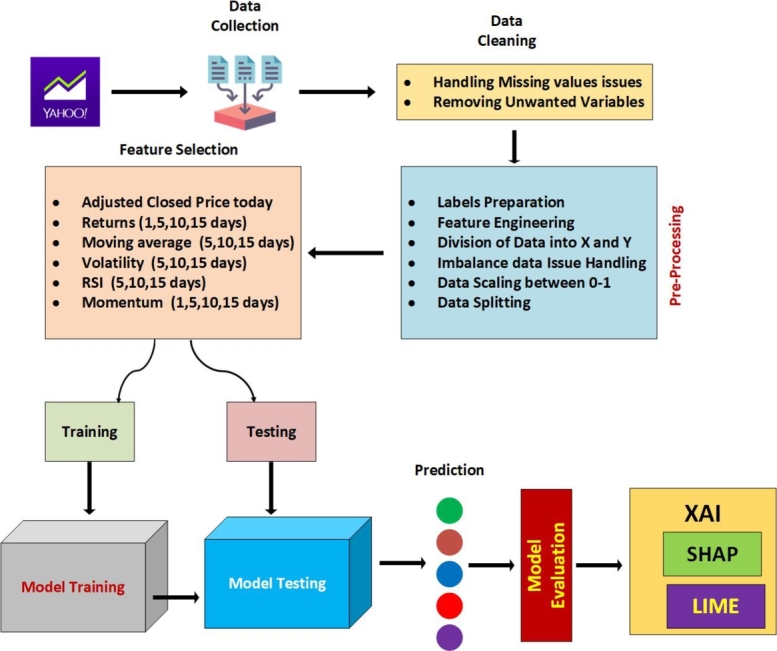
Workflow of the Proposed Methodology.

The study used the following methodology. The data from Yahoo Finance [40] was downloaded for four stock markets namely S&P500, DAX30, Nikie225 and FTSE. The raw data underwent preprocessing for quality enhancement, followed by feature engineering to create new features. Subsequently, the performance of both our proposed model and benchmark models was evaluated using the four datasets. Finally, SHAP and LIME methodologies were used to gain insights into the contribution of individual features to the model's predictions and to offer locally understandable explanations for individual forecasts, respectively. The roadmap of the research is depicted in Fig. 2. Various stages of the process are explained as follows.

### 3.1 Data collection

Yahoo! Finance is the source of data for this study. The adjusted closing prices data for the time period 1990−2022 of different stock markets (DAX30, FTSE100, Nikkie225 and S&P500) are obtained from Yahoo! Finance [40]. The period from 1990−2022 was selected to provide a comprehensive dataset that spans multiple market cycles, economic crises, and significant global events. This 32-year period includes key events such as the dot-com bubble [41], [42], the 2008 financial crisis [43], and the COVID-19 pandemic [44], allowing the model to learn from diverse market conditions.

The time frame also captures the evolution of technology and trading practices, reflecting how these changes have impacted market behaviour. By covering both stable and volatile periods, this duration ensures that the model can detect long-term trends and generalize better across different economic environments, improving the robustness and accuracy of stock trend predictions. The basic statistics of the data is reported in Table 2.

**Table 2:** Overview of the datasets used in the study.

Dataset	Duration	No. of Observations
DAX30	1990-2022	613
FTSE100	1990-2022	621
S&P500	1990-2022	656
Nikkie225	1990-2022	644

The selection of the four stock markets—S&P500, DAX30, Nikkei225, and FTSE—was based on their global significance, market diversity, and ability to represent different economic regions. These indices were chosen to ensure that our model is tested across a wide range of market conditions, thereby enhancing its robustness and generalizability.

• **Standards and Poor's (S&P500)** As one of the most prominent stock market indices, the S&P500 represents the performance of 500 large companies listed on U.S. stock exchanges. It is a critical benchmark for the U.S. economy, which is one of the largest and most influential markets globally. The S&P500 captures a wide range of industries, providing a broad representation of market sentiment and economic health, making it an essential dataset for testing financial models.
• **Deutscher Aktienindex (DAX30):** The DAX30 index includes 30 major companies traded on the Frankfurt Stock Exchange, representing the largest and most liquid stocks in Germany. Germany is the largest economy in Europe, and the DAX30 serves as a key indicator of the European market's health and performance. Including DAX30 ensures our model captures trends in the European market, providing geographic diversification to our dataset.
• **Nikkei225:** The Nikkei225 is Japan's leading stock index and tracks the performance of 225 prominent companies on the Tokyo Stock Exchange. Japan has the third-largest economy in the world, and the Nikkei225 is a crucial gauge of market trends in Asia. Including this index allows our model to capture trends in a major Asian market, thus covering a diverse range of market dynamics.
• **FTSE100:** The FTSE100 index comprises the 100 largest companies listed on the London Stock Exchange and is a key indicator of the UK's economic performance. As one of the largest financial hubs globally, the UK's inclusion through the FTSE100 provides insights into market trends in a post-Brexit economy, further enhancing the geographic and economic diversity of the datasets used.

By including these four major stock indices, we ensure that our proposed model (DNN) is trained and tested on data representing the U.S., European, Asian, and UK markets, covering a wide range of economic conditions, market behaviours, and industrial sectors. This diversity is critical for developing a model that can generalize well across different market environments and improve its real-world applicability.

### 3.2 Pre-processing

Pre-processing of data is an important step which transforms the raw, unorganized, and unstructured financial time series data for better learning of the model and is carried out as follows:

#### 3.2.1 Data labelling

In data preprocessing for supervised learning, data labelling is an indispensable part of the process. In this work, we prepared five different labels (trends) from the historical data namely, upward, downward, double top, rounded bottom, and rounded top [18]. To enhance the granularity of our analysis, we chose a 15-days window. If any trend is identified at any time point, we consider the preceding 15 days windows as features for the particular trend. This time frame was selected after careful consideration of trade-offs between shorter and longer window lengths.

Shorter windows (e.g., 5-10 days) may capture more immediate price fluctuations but tend to introduce noise, making it harder to detect more stable, meaningful trends. These shorter windows might be too sensitive to minor price changes that do not reflect real market trends, thus increasing the likelihood of false signals. On the other hand, longer windows (e.g., 20-30 days) can smooth out noise but at the cost of responsiveness, as they might fail to capture faster, short-term trends that are often critical in dynamic financial markets.

The 15-day window strikes a balance between these extremes, allowing the model to capture trends that reflect significant market movements without being too short to get distracted by noise or too long to overlook important short-term trends. This time frame aligns with common practices in financial analysis, where short- to medium-term trends are often considered the most actionable for trading and investment strategies. Empirical testing with varying window lengths (e.g., 10, 20, and 30 days) showed that the 15-day window produced more reliable and accurate trend predictions across our target labels, as evaluated using accuracy, precision, and F1-score.

Therefore, the choice of the 15-day window optimally balances granularity, responsiveness, and stability in trend detection, ensuring that the model remains sensitive to meaningful market patterns while avoiding overreaction to minor fluctuations.

#### 3.2.2 Addressing class imbalance

After labelling, we observed that the dataset is facing the challenge of imbalanced class/label distribution. The presence of disparate class frequencies within the dataset poses a significant hurdle, potentially biasing the results of our analyses. To address the imbalance classes issue, we employed the Synthetic Minority Over-sampling Technique (SMOTE) as a remedial strategy. SMOTE [45], is a widely recognized technique designed to reduce imbalances by artificially augmenting minority class instances. This method generates synthetic instances in the feature space, thereby levelling the class distribution and fortifying the model against the adverse effects of class imbalance.

While SMOTE is effective in balancing class distributions, it is important to manage potential risks such as the introduction of noise or overfitting when the minority class is highly underrepresented. To mitigate these risks, we carefully tuned the SMOTE parameters and combined this technique with regularization methods (*Dropout*), ensuring the model generalizes well to unseen data. This careful balance ensures that SMOTE remains a powerful tool for improving model performance while preserving its ability to accurately predict trends in real-world scenarios.

### 3.3 Feature engineering

Our original data contained only the closing prices which were not sufficient for predicting the complex trends. Therefore, we used feature engineering to create new features (returns, moving average, volatility, relative strength index and momentum) for use as features with the closing prices. The formulae for these features are given below.

1. Returns: The stock returns [46] are used to record the daily changes in the stock market given as in Equation (1);
  (1) $$R=\frac{P_{f}-P_{i}}{P_{i}}$$
  Where $P_{i}$ and $P_{f}$ are the prices at the start and end of a time period. We calculated returns for 1, 5, 10 and 15 days.
2. Moving Average: It is a measure to record the trends direction in financial market in the last *n* days [47], as shown in Equation (2).
  (2) $$MA(n)=\frac{1}{n}\sum_{i=t-n+1}^{t}P_{i}$$
  Note that MA(n) is the moving average of last *n* days, and $P_{i}$ is the closing price on day *i*. We calculated moving average for 5, 10 and 15 days.
3. Volatility: The percent fluctuation of the stock market is recorded through statistical counter for stock, as given by [48] and presented in Equation (3).
  (3) $$V=\sqrt{\text{Variance}}\cdot \sqrt{\text{No. of days}}$$
  Here,“Variance” represents the variance of the price of last *n* of days. We calculated *V* for 5, 10 and 15 days.
4. Relative Strength Index: This is a momentum based indicator depicting the fluctuations technically given by [49], as depicted in Equation (4);
  (4) $$RSI=100-\frac{100}{1+RS}$$
  Where RS is the relative strength, which is the average of *n* days' up closes divided by the average of *n* days' down closes. We calculated *RSI* for 5, 10 and 15 days.
5. Momentum: The rate of change in the price of security of the market is depicted in the momentum [50] and demonstrated in Equation (5)
  (5) $$M=\frac{\text{Closing Price Today}-\text{ Closing Price}\;\text{N}\;\text{Days Ago}}{\text{ Closing Price}\;\text{N}\;\text{Days Ago}}⁎100$$
  Momentum is calculated for 1, 5, 10 and 15 days.

For each day we obtain 18 features namely, closing price, returns (4 values), moving average (3 values), volatility (3 values), relative strength index (3 values) and momentum (4 values). Therefore, for a 15 days time window, our input vector contains a total of 270 values.

### 3.4 *K* fold cross validation

The dataset was divided into K folds using the *K*-fold cross-validation technique, where K=10. Each fold served as the testing set once, while the training and testing procedures were iteratively executed 10 times. This choice of K=10 is commonly used because it provides a good balance between bias and variance. By iterating 10 times, each data point is included in the testing set exactly once, while the remaining data points are used for training in each iteration.

The use of 10-fold cross-validation offers a robust evaluation of model performance by systematically rotating through several folds, ensuring that the model is exposed to different subsets of the data. This reduces the effect of data randomness, prevents overfitting, and provides more reliable insights into the model's capacity to generalize to new, unseen data. Additionally, performing this process 10 times helps mitigate the risk of any single fold being an outlier, thus ensuring a more consistent and reliable estimation of the model's performance.

### 3.5 The proposed model

In this study, we propose a sequential deep learning model to predict stock market trends. The architecture, comprising 6 layers, was chosen based on extensive experimentation using grid search and cross-validation to balance model complexity, performance, and generalization. The architecture consists of one input layer, four hidden layers, and one output layer, with neurons allocated in each layer as follows: 270, 135, 67, 405, 200, and one, respectively.

The number of neurons in the input layer (270) was chosen to match the dimensionality of the input data (features). The first hidden layer has 135 neurons, and subsequent layers follow a pattern of reduction (67 neurons) followed by an increase in neuron count (405 neurons) to allow the model to progressively reduce complexity, then expand its learning capacity. This progression was found to improve feature abstraction and allow the model to learn both low-level and high-level features effectively. The final hidden layer with 200 neurons refines these learned representations before passing them to the output layer.

The activation function used in the input and the first three hidden layers is ReLU (Rectified Linear Unit), chosen for its ability to accelerate convergence by mitigating the vanishing gradient problem. The fourth hidden layer uses a uniform activation function, helping regularize the model and reduce overfitting. To further prevent overfitting, a *Dropout* layer was added after the first hidden layer, ensuring that the network learns robust features by randomly deactivating a fraction of neurons during training. The kernel initializer in these layers was set to *random_normal* to initialize the weights, ensuring that training begins with a good weight distribution and avoids issues like symmetry during learning [51], [52].

The output layer employs the softmax activation function, which is appropriate for multi-class classification, converting raw outputs into probability distributions over the five trend classes. The choice of softmax enables the model to make confident predictions about which class the input data belongs to.

To optimize the model, we used the Adam optimizer, chosen for its adaptive learning rate properties, which ensure faster convergence while maintaining stability during training. The loss function selected is categorical cross-entropy, which is commonly used for multi-class classification as it effectively measures the divergence between the predicted class probabilities and the actual labels.

The number of layers, neurons, and activation functions were fine-tuned through iterative experimentation, evaluating model performance across multiple configurations. Grid search and cross-validation were used to determine the best performing architecture while preventing overfitting. Finally, we implemented 10-fold cross-validation to rigorously evaluate the model's generalization and robustness across accuracy, precision, recall, and F1-score, ensuring balanced performance across all metrics.

### 3.6 Benchmark algorithms

#### 3.6.1 Logistic regression (LR)

Logistic Regression (LR) [53], [54] is a machine learning technique frequently utilized in classification problems. The mathematical function of LR is as outlined in Equation (6).

(6) $$h_{\theta }(X)=1/1+e^{-(\beta_{0}+\beta_{1}X)}$$

Here *θ* are the parameter values that needs to be learned and *X* represents the feature set. In this study, we used the scikit-learn library. Subsequently, we set up the class for multi-label classification whereby the “passed-out argument: multinomial” and “solver: ibfgs” is utilized for the multi class classification.

#### 3.6.2 Random forest (RF)

Random Forest (RF) [55], [56] is a machine learning technique employed for binary and multi-class classification problems. RF evolved from decision trees constituting a massive number of trees running discretely to predict the positive label. We trained the classifier on several parameters such that number of estimators, random state, and criterion in order to attain a required accuracy. To classify the positive label, we demarcated the estimators (500) as no. of trees, applied the loss function of entropy and utilized 10-fold cross validation to evaluate the model.

#### 3.6.3 Support vector machine (*SVM*)

Support Vector Machine (SVM) [57], [58] is a well known technique of supervised machine learning suited for binary and multi-class classification. The purpose of this method is to identify an optimal boundary within feasible outcomes. We employed decision function “ovo” to train the model on the datasets.

### 3.7 Evaluation metrics

As basis for our evaluation, we calculated confusion matrix, and further based on the confusion matrix Accuracy, Recall, Precision and F1-score were calculated. For formulae, refer to Table 3.

• **Accuracy** measures the proportion of correctly classified instances (both true positives and true negatives) over the total number of instances, giving an overall performance measure.
• **Precision** quantifies the proportion of correctly predicted positive instances out of all instances predicted as positive, indicating the model's ability to minimize false positives.
• **Recall** reflects the proportion of actual positive instances that were correctly identified by the model, capturing the model's ability to detect true positives.
• **F1-score** is the harmonic mean of Precision and Recall, providing a balanced measure when there is an uneven class distribution or when both false positives and false negatives need to be minimized.

**Table 3:** Evaluation metrics.

Name	Formula
Accuracy	$\frac{TP+TN}{TP+TN+FP+FN}$
Recall	$\frac{TP}{TP+FN}$
Precision	$\frac{TP}{TP+FP}$
F1-Score	$2\frac{Precision\cdot Recall}{Precision+Recall}$

Note: *TP*, *TN*, *FP*, and *FN* represent True Positive, True Negative, False Positive, and False Negative, respectively.

### 3.8 SHAP

The SHAP model [19], [20], [21], [22], [59], which has roots in cooperative game theory, offers a framework to determine the contribution of each input attribute to the predictions made by the algorithm. The Shapley value, a measurement of each feature's marginal contribution to the prediction outcome, is assigned to each feature by SHAP, an additive feature attribution approach. When all possible permutations of the feature values are considered, the Shapley value represents a fair distribution of the overall forecast across features. The Shapley value for a specific feature in the context of a specific prediction can be written mathematically as shown in Equation (7):

(7) $$\phi_{i}(f,x)=\sum_{S\subseteq N_{i}}\frac{|S|!\cdot (N-|S|-1)!}{|N|!}[f(x_{s\cup \{x_{i}\}})-f(x_{s})]$$

Where: *f* represents the prediction model, *x* is the input feature vector, *N* is the set of features, *S* is a subset of features excluding the feature *i* i.e., feature under XAI analysis, $x_{s}$ is the feature vector with features in subset *S* replaced by baseline values and $x_{i}$ is the value of feature *i* in the input vector *x*.

### 3.9 LIME

To provide locally understandable explanations for individual forecasts, we incorporated the LIME system [23], [24], [25], [26], [60] which is a powerful method to reveal the local decision-making process of complex machine learning models. LIME is based on a model-agnostic approach which focuses on explaining specific predictions, rather than interpreting the model as whole. This is achieved by approximating the model's behaviour using a more understandable “surrogate” model. LIME's core methodology involves perturbing a prediction instance's input features and tracking changes in the model's output. The dataset produced by these perturbations was used to train the surrogate model, which successfully imitated the behaviour of the original model close to the instance of interest. Mathematically, LIME minimizes the following objective function to mathematically approximate the local reason for a given prediction, as presented in Equation (8).

(8) $$L(f^{\prime },\pi_{x})=\sum_{x^{\prime }\in \pi_{x}}{\left(f(x^{\prime })-f^{\prime }(x^{\prime })\right)}^{2}+\Omega (f^{\prime })$$

Where $f^{\prime }$ represents the surrogate interpretable model, *f* is the complex model, $\pi_{x}$ denotes the perturbed instances around *x* (the input feature vector), $x^{\prime }$ is a perturbed instance from $\pi_{x}$ and Ω is a regularization term ensuring simplicity and interpretability of the surrogate model.

## 4 Results and discussion

We report the performance of our proposed and benchmark models based on accuracy, precision, recall and F1-score. Furthermore, recognizing the growing interest in model interpretability, we used SHAP and LIME techniques to investigate the model's decision-making rationale as well.

**Figure 3:**
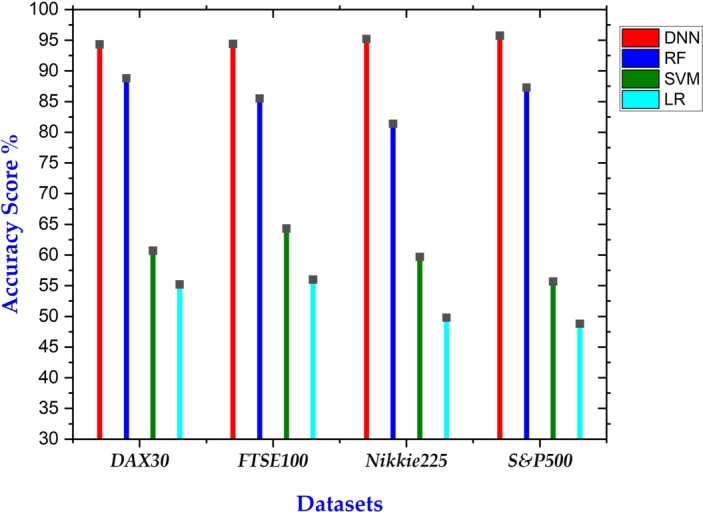
Summary of Accuracy on all 4 datasets.

The performance metrics of both the proposed and benchmark models, focusing on accuracy, are detailed in Table 4. Across all four datasets, our proposed model consistently outperforms the benchmark algorithms in terms of accuracy. Specifically, the average accuracy of our proposed model stands at 94.9%, representing an improvement of at least 10% over the next best-performing model, which is random forest algorithm with an average accuracy of 85.75%. The performance of all models shows relatively stable trends across the datasets, without significant fluctuations. Importantly, the standard deviation in the accuracy of our proposed model across the four datasets is the smallest among all models. A graphical summary of the results is depicted in Fig. 3.

**Table 4:** Overall accuracy scores of the proposed model and benchmark models on four datasets.

Dataset	DNN	RF	SVM	LR
DAX30	94.31	88.80	60.70	55.20
FTSE100	94.40	85.50	64.30	56.00
Nikkei225	95.21	81.4	59.60	49.80
S&P500	95.74	87.30	55.70	48.80

Note: Acronyms: LR – Logistic Regression, SVM – Support Vector Machine, RF – Random Forest, DNN – Deep Neural Network.

**Figure 4:**
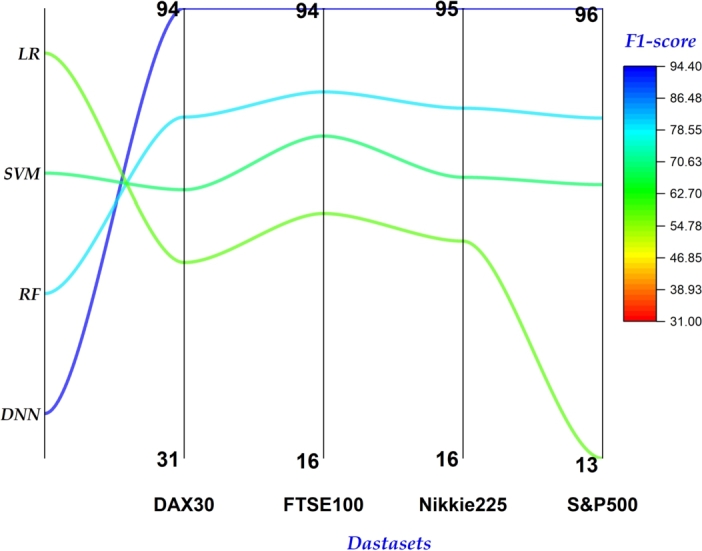
Summary of F1 Scores on all 4 datasets.

**Figure 5:**
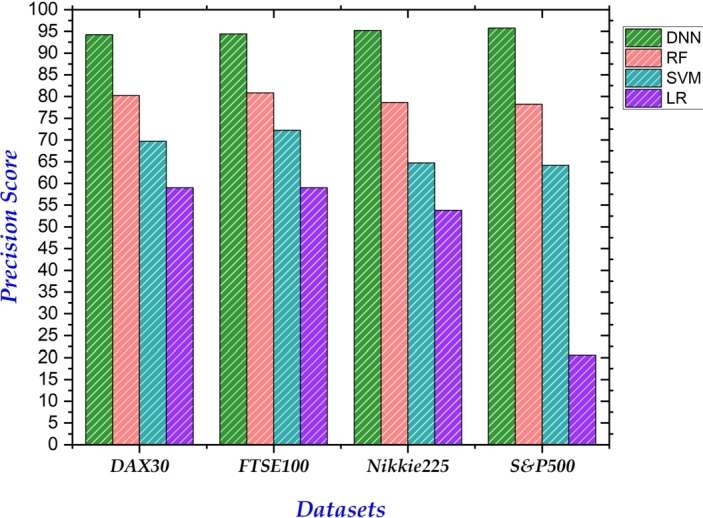
Summary of Precision Scores on all 4 datasets.

**Figure 6:**
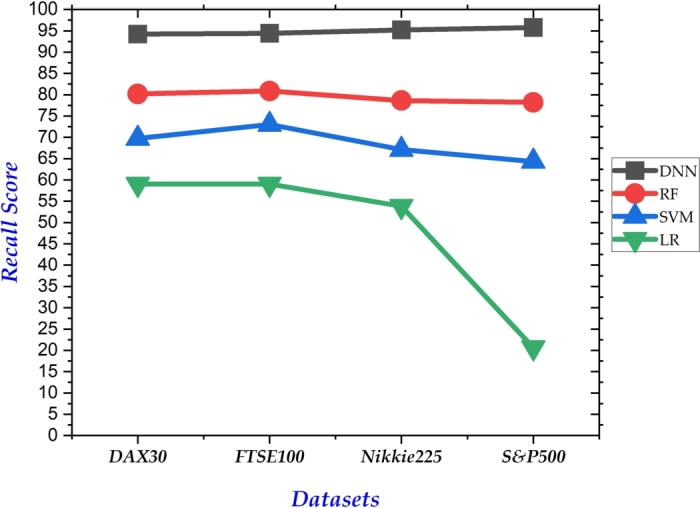
Summary of Recall Scores on all 4 datasets.

In classification problems, accuracy alone can be deceptive, neglecting false positives/negatives, and sensitivity to thresholds. Considering precision, recall, and F1 score provides a betters understanding of a model's ability to correctly identify and differentiate classes. Therefore, we also report results of our proposed and benchmark models on these measures. Fig. 5 depicts the precision achieved by each model across the four datasets. Notably, our proposed model consistently outperformed all others, achieving the highest precision on each dataset. Similarly, our model exhibited superior performance in both recall (Fig. 6) and F1-score (Fig. 4). In both metrics, our approach achieved the highest values across all datasets, reinforcing its effectiveness in correctly identifying and differentiating between classes.

The effectiveness of the proposed model to handle multi-label classification tasks effectively may possibly be attributed to its architecture which heavily relies on neurons interconnections as well as ‘drop out’ for avoiding the overfitting problem. The RF model performs well due to its ensemble approach, while LR and SVM, which have simpler structures, show less impressive predictive abilities.

**Figure 7:**
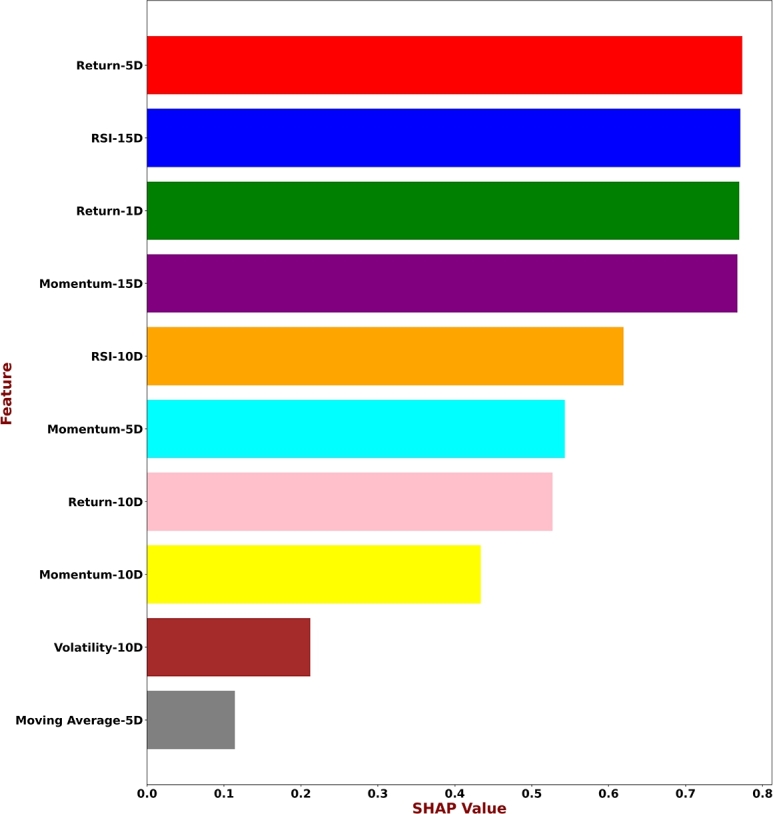
Top 10 contributory features explanation by SHAP.

**Figure 8:**
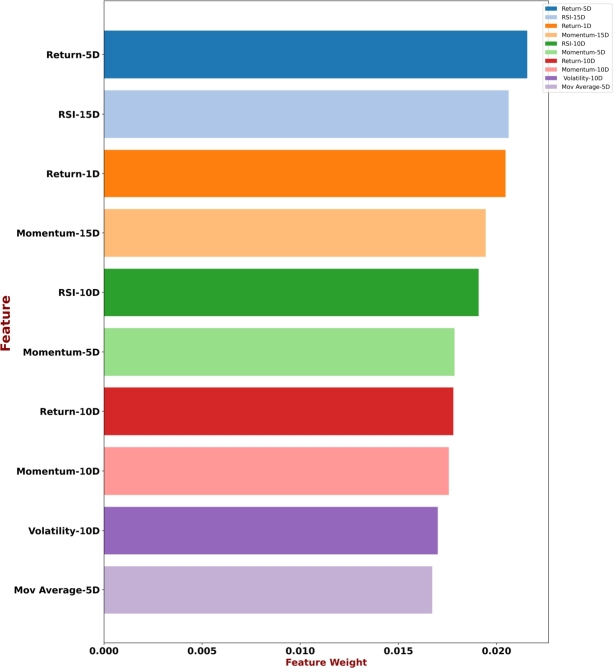
Feature weights explanation by LIME.

In terms of explainability, the SHAP model stands out from other attribution strategies because of its unique capacity to offer a coherent and consistent explanation of predictions. To highlight the complex connections between input features and prediction outcomes, this study applied SHAP to our proposed model, the results of which are summarized in the Fig. 7. The goal is to provide stakeholders with a more transparent and perceptive decision-making process for stock market analysis. This step enables investors and analysts to recognize the important factors influencing the model's outcomes, facilitating well-informed decision-making. To understand the dynamics of individual projections and help stakeholders, we also applied LIME to our proposed model (Fig. 8). By complementing SHAP with LIME, we were able to identify how input features shape specific predictions, leading to understand the reasoning behind each decision/prediction. The LIME model's emphasis on local interpretability provides a practical approach for analyzing the reasoning behind complex stock market prediction models.

Following the application of SHAP and LIME approaches, the top 10 features were identified. Subsequently, the remaining features were discarded and the proposed model was re-trained with the top 10 features. The performance on various evaluation criterion is reported in Table 5.

**Table 5:** Overall result scores of the proposed model on top ten contributory features.

Dataset	Accuracy	Precision	Recall	F1-score
DAX30	83.68	98.28	100	99.13
FTSE	82.00	96.15	100	98.04
S&P500	77.32	95.04	96.29	95.04
Nikkie225	75.28	92.86	99.21	95.93

Comparing the performance of our proposed model reveals interesting trade-offs between using all features and selecting only the top 10 most contributory ones. All datasets show a decrease in accuracy when using only the top 10 features. The largest drop is in the Nikkei dataset, followed by S&P500, FTSE and DAX30 respectively. Interestingly, we observed that precision and recall increases for all datasets with the top 10 features. F1-score generally increases with the top 10 features. The highest increase is in DAX30, followed by Nikkei. The decrease in accuracy while other scores (precision, recall, F1-score) increased when using only the top 10 features identified by SHAP and LIME is an interesting phenomenon. There can be several possible explanation for this. For instance, selecting only the top 10 features based on methods like SHAP and LIME might discard valuable information contained in other features. Even if those features have smaller individual contributions, they might interact with each other or with the target variable in ways that SHAP and LIME do not fully capture. This loss of information can negatively impact the model's ability to accurately predict unseen data, leading to lower accuracy.

### 4.1 Discussion

Our study shows that the proposed deep learning model, enhanced with explainable AI (XAI) techniques, provides a robust and interpretable method for predicting multi-class stock market trends. Tested on four datasets—S&P500, DAX30, FTSE100, and Nikkei225—the model consistently outperformed traditional machine learning approaches like RF, SVM, and LR, demonstrating its ability to capture complex non-linear patterns in financial data.

The proposed framework extends beyond the capabilities of conventional approaches in several ways [8], [11], [7], [27], [12], [14], [15], [28], [29], [30], [33], [31], [32]. Prior research has predominantly focused on binary classification and price prediction, often neglecting the multi-class trend prediction that is crucial for capturing the complexity of financial markets. For instance, studies employing logistic regression or support vector machines have shown limited efficacy in dealing with multi-class classifications and sequential dependencies in stock data. In contrast, the DNN architecture presented here, with its deep layers and dropout regularization, effectively addresses these challenges, achieving superior predictive performance. Additionally, the integration of XAI techniques sets this study apart from existing models. Previous works, such as those using gradient boosting or other tree-based models [34], [35], have incorporated XAI to a limited extent but focused mainly on binary or price-based predictions. This study, however, demonstrates how XAI can be effectively applied to multi-class predictions, providing both high accuracy and interpretability.

### 4.2 Implications

The deployment of this model in financial decision-making systems holds significant potential. By providing both accurate predictions and explainable outputs, the model can support portfolio management, risk assessment, and investment strategy development. Its predictive power and transparency enable more informed decision-making for investors, allowing for better risk management and strategic planning in dynamic market environments. The integration of XAI tools, such as SHAP and LIME, further strengthens trust in AI-driven systems by offering clear insights into the factors driving predictions. This transparency builds confidence among investors and analysts, making the model a more viable tool for real-world financial applications, whether in long-term investments or more immediate trading decisions.

## 5 Conclusions

This study proposed a novel deep learning framework, augmented with explainable AI (XAI) techniques, for the prediction of multiple stock market trends. By classifying five distinct trends—upward, downward, double top, rounded bottom, and rounded top—the model addresses a critical gap in the literature where multi-class trend prediction has been under-explored. The proposed DNN model consistently outperforms traditional machine learning techniques, such as SVM and RF, across four major financial indices, demonstrating superior predictive accuracy, precision, recall, and F1-score. The integration of XAI methods, namely SHAP and LIME, enhances the interpretability of the model's predictions, offering both global and local explanations of key factors driving the stock market trends. This interpretability not only improves trust in the model's predictions but also provides stakeholders with actionable insights for better financial decision-making.

### 5.1 Limitations and future research directions

Despite the promising results, the proposed model faces several limitations. First, while the model performs well across historical datasets, its generalizability in real-time trading environments remains untested. Future research should focus on incorporating external data sources such as social media sentiment, economic indicators, and real-time news feeds. These additions would enable the model to capture a broader range of factors influencing market trends, potentially improving its predictive power during volatile or crisis-driven periods. Furthermore, refining the model to accommodate real-time data streaming is essential for applications in high-frequency and algorithmic trading systems, allowing for dynamic and immediate predictions. Furthermore, while SHAP and LIME improve interpretability, there remains a risk of oversimplifying the complex relationships between features in financial markets. Continued refinement of these XAI techniques is necessary to ensure that critical subtleties are not lost in translation. The use of advanced interpretability methods that account for interactions between features could provide even greater insights into the decision-making processes of deep learning models.

#### Data and code availability

The data and code are available upon request from the corresponding author.

## CRediT authorship contribution statement

**Dost Muhammad:** Writing – original draft, Visualization, Validation, Software, Methodology, Investigation, Funding acquisition, Formal analysis, Data curation, Conceptualization. **Iftikhar Ahmed:** Writing – review & editing, Supervision, Project administration, Methodology, Investigation, Conceptualization. **Khwaja Naveed:** Writing – review & editing, Writing – original draft, Validation, Methodology. **Malika Bendechache:** Writing – review & editing, Supervision, Methodology, Investigation, Formal analysis.

## Declaration of Competing Interest

The authors declare that they have no known competing financial interests or personal relationships that could have appeared to influence the work reported in this paper.
